# Decreased Serum Hepatocyte Growth Factor (HGF) in Autistic Children with Severe Gastrointestinal Disease

**DOI:** 10.4137/bmi.s3656

**Published:** 2009-11-27

**Authors:** A.J. Russo, A. Krigsman, B. Jepson, Andrew Wakefield

**Affiliations:** 1 Research Director, Health Research Institute/Pfeiffer Treatment Center, 4575 Weaver Parkway, Warrenville, Illinois 60555, USA; 2 Thoughtful House Center for Children, 3001 Bee Caves Road, Austin, Texas, 78746, USA. Email: ajrusso@hriptc.org

**Keywords:** autism, hepatocyte growth factor, GI disease, met gene

## Abstract

**Aim:**

To assess serum Hepatocyte Growth Factor (HGF) levels in autistic children with severe gastrointestinal (GI) disease and to test the hypothesis that there is a relationship between GI pathology and HGF concentration.

**Subjects and Methods:**

Serum from 29 autistic children with chronic digestive disease (symptoms for a minimum of 6–12 months), most with ileo-colonic lymphoid nodular hyperplasia (LNH—markedly enlarged lymphoid nodules) and inflammation of the colorectum, small bowel and/or stomach), and 31 controls (11 age matched autistic children with no GI disease, 11 age matched non autistic children without GI disease and 9 age matched non autistic children with GI disease) were tested for HGF using ELISAs. HGF concentration of autistic children with GI disease was compared to GI disease severity.

**Results:**

Autistic children with GI disease had significantly lower serum levels of HGF compared to controls (autistic without GI disease; p = 0.0005, non autistic with no GI disease; p = 0.0001, and non autistic with GI disease; p = 0.001). Collectively, all autistic children had significantly lower HGF levels when compared to non autistic children (p < 0.0001). We did not find any relationship between severity of GI disease and HGF concentration in autistic children with GI disease.

**Discussion:**

These results suggest an association between HGF serum levels and the presence of GI disease in autistic children and explain a potential functional connection between the Met gene and autism. The concentration of serum HGF may be a useful biomarker for autistic children, especially those with severe GI disease.

## Introduction

Autism is a complex, behaviorally defined neurodevelopmental disorder characterized by social deficits, language impairments, and repetitive behaviors with restricted interests. There has been a dramatic increase in the diagnosis of autism over the past decade.^1^,^2^

The etiology of this complex disease is highly heritable, but likely involves environmental factors.^58^ Twin studies demonstrate concordance rates of 82%–92% in monozygotic twins and 1%–10% concordance rate in dizygotic twins.^3^ Sibling recurrence risk (6%–8%) is 35 times the population prevalence.^1^,^4^

Genetic analysis suggests that as many as 15 genes might be involved in Autism Spectrum Disorders (ASD), including variants on chromosomes 2q, 7q, 15q, and 17q.^5^–^8^

Children with autistic spectrum disorders (ASD) frequently have accompanying gastrointestinal, immunological, or nonspecific neurological symptoms.^9^–^15^

Based on neurobiological findings and location within a chromosome 7q31 autism candidate gene region,^62^ Campbell et al analyzed the MET receptor tyrosine kinase gene in a family based study of autism and found a functional variant of MET with autism with a calculated relative risk of 2.27.^16^

MET, found originally to be involved in cancer metastasis,^42^ facilitates the signaling of hepatocyte growth factor (HGF)/scatter factor and its involvement in peripheral organ development and repair,^17^–^19^ immune function^20^–^22^,^61^ and gastrointestinal repair.^18^,^23^,^24^,^59^

In the central nervous system, MET contributes to development of the cerebral cortex^25^,^26^ and cerebellum.^25^ Abnormal MET/HGF signaling in the cerebral cortex results in atypical interneuron migration from the ganglionic eminence and reduced interneurons in the frontal and parietal regions of cortex.^28^,^29^ In the cerebellum, aberrant signaling causes decreased proliferation of granule cells and reduction in the cerebellar size, particularly in the vermis.^25^ Both of these aberrations are consistent with those observed in brains of individuals with autism.^26^,^27^,^60^

Hepatocyte growth factor (HGF), an 82 kDa, 674 amino acid residue heterodimeric glycoprotein, was originally isolated from rat platelets.^32^,^33^ This growth factor has also been called scatter factor, hepatopoietin A, and mammary growth factor.^34^ It is one of a small family of factors lacking significant homology with other known growth factors, but including an HGF-like factor known as macrophage stimulating protein (MSP).^35^–^38^ HGF has mitogenic, morphogenic, and motogenic effects on hepatocytes, as well as endothelial, mesenchymal and hematopoietic cell types,^37^,^39^,^40^ and demonstrates noticeable species cross-reactivity.^41^

Children with autistic spectrum disorders frequently have accompanying gastrointestinal symptoms^49^–^51^ and pathology, which includes inflammation of the GI tract^53^–^56^ and autoimmunity related to GI disease severity.^57^ Because a MET variant exists in the genome of a significant number of autistic individuals, we hypothesized that this might result in abnormal levels of serum HGF, particularly those with severe GI disease.

## Materials and Methods

### ELISA to measure serum HGF (ELISA kit, R&D systems, minneapolis, minn.)

All reagents and specimens were equilibrated to room temperature before the assay was performed. A 1:51 dilution of the patient samples was prepared by mixing 10 μl of the patient’s sera with 0.5 ml of Serum Diluent. One hundred microliters of calibrators (20–200 Eu/ml antibodies), positive and Negative control serums, serum diluent alone, and diluted patient samples were added to the appropriate microwells of a microculture plate (each well contained affinity purified polyclonal IgG to HGF). Wells were incubated for 60 minutes (±5 min) at room temperature, then washed 4x with wash buffer. One hundred microliters of pre-diluter anti-human IgG conjugated with HRP was added to all microwells, incubated for 30 minutes (±5 min) at room temperature, then wash 4x with wash buffer. One hundred microliters of enzyme substrate was added to each microwell. After approximately 30 minutes at room temperature, the reaction was stopped by adding 50 μl of 1 M sulfuric acid, then the wells were read at 405 nm with an ELISA reader (BioRad Laboratories, Inc., Hercules, CA, USA).

### Subjects and scoring of severity of GI disease

Serum from autistic individuals with GI disease was obtained from the Thoughtful House, Austin, Texas.^a^ All 29 children in this study with ASD (median age 6 years; range 2–16; 34 male) with gastrointestinal symptoms, were investigated by ileo-colonoscopy. Macroscopic and histological features of the upper and lower GI tract were scored. A reproducible scoring system, similar to the Crohn’s Disease Endoscopic Index of Severity (CDEIS) was developed and used to evaluate this unique type of observed enterocolitis and severity of disease (particularly inflammation). Patients were scored according to mild (1 point), moderate (2 points) and marked (3 points) disease in each area (upper and lower GI) and for scope (macroscopic) and histology of each area. Therefore, the maximum score for GI disease was 12 (3 points each for upper scope, upper histology, lower scope and lower histology). A point system was also developed for severity of lymphoid nodular hyperplasia (LNH). Patients were scored according to mild (1 point), moderate (2 points) and marked (3 points) LNH in each area (upper and lower GI) for a maximum of 6 points. And finally, a point system was also developed for severity of erythema. Patients were scored according to mild (1 point), moderate (2 points) and marked (3 points) erythema in each area (upper and lower GI) for a maximum of 6 points.

### Controls

Three control groups (total n = 31) were studied (11 age matched autistic children with no GI disease, 11 age matched non autistic children without GI disease and 9 age matched non autistic children with GI disease) (mean 71 months), gender (78% male) and diagnosis (55% of autistic controls had regressive onset). Serum and medical history of controls were obtained from the Autism Genetic Resource Exchange—AGRE.^b^ Patients were categorized as having GI disease, not having GI disease, or no autistic based on the medical history provided by AGRE.

#### Informed consent

Informed consent was obtained from all patients in this study by the Thoughtful House and AGRE, where appropriate. IRB of the Thoughtful House approved this project.

### Serums

Experimental (Thoughtful House) and control (AGRE) serums were morning draws, then frozen at −70 C immediately after collection and cell/serum separation, then stored at −70 C until thawed for use in ELISAs.

### Statistics

Inferential statistics were derived from unpaired t-test and odds ratios with 95% confidence intervals. ANOVA analysis was used to do an analysis of variance and multiple comparisons.

## Results

Serum from 29 autistic children with chronic digestive disease (most with ileo-colonic lymphoid nodular hyperplasia (LNH) and inflammation of the colorectum, small bowel and/or stomach), and 31 controls (11 age matched autistic children with no GI disease, 11 age matched non autistic children without GI disease and 9 age matched non autistic children with GI disease) were tested for HGF using an ELISA designed to quantitate HGF levels (described above). Each assay was repeated two or more times, with multiple wells for each serum in each assay. The results of a typical assay are summarized on Figures 2 and 3 (Fig. 2—standards; Fig. 3—control/experimental serums).

**Figure 1.**
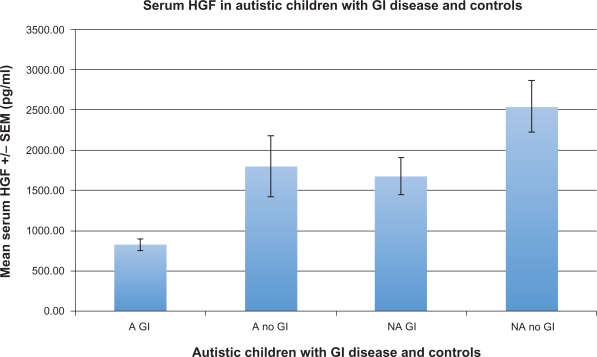
The mean +/− SD HG F concentration (pg/ml) of 29 autistic children with chronic digestive disease (most with ileo-colonic lymphoid nodular hyperplasia (LNH) and inflammation of the colorectum, small bowel and/or stomach) (A GI), and 30 controls (11 age matched autistic children with no GI disease (A No GI), 11 age matched non autistic children without GI disease (NA no GI) and 9 age matched non autistic children with GI disease (NA GI)).

**Figure 2.**
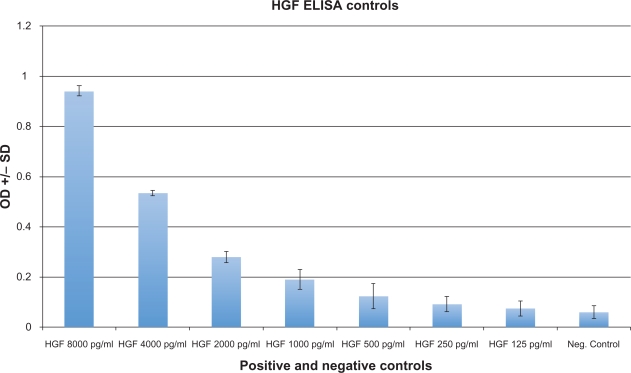
HGF serum concentration was established for each individual by testing and correlating to known standards of various concentrations of MPO (8000 pg/ml–125 pg/ml), as well as negative control (serum diluent alone).

**Figure 3.**
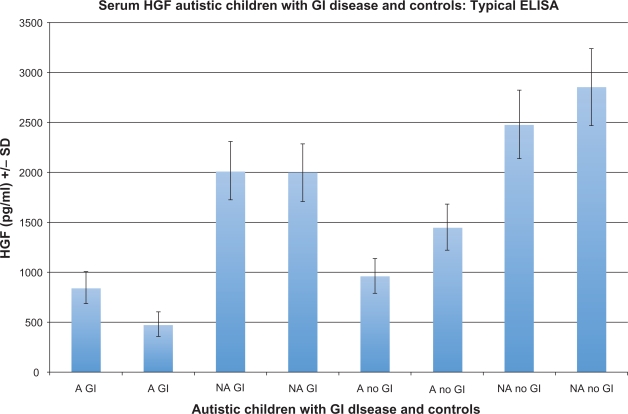
Serum HGF concentration was measured in a typical ELISA. Two autistic children with GI disease (A GI), 2 non autistic children with GI disease controls (NA GI), 2 autistic children with no GI disease (A No GI) and 2 non autistic children with no GI disease controls (NA no GI) were tested. Four replicate samples were tested for each individual.

**Figure 4.**
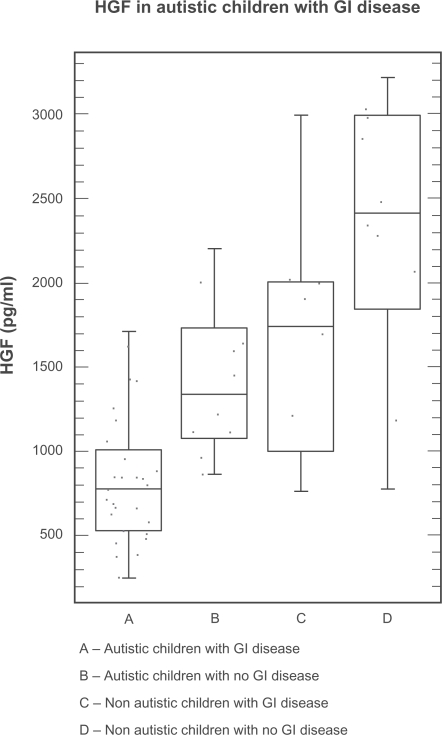
A Box Plot representation of HGF levels showing variability in autistic and non autistic groups (with and without GI disease).

Serum HGF levels of autistic children with GI disease were significantly lower than all non autistic controls (p < 0.0001) (Table 3), as well each of the controls (autistic without GI disease; p = 0.0005, non autistic with no GI disease; p = 0.0001, and non autistic with GI disease; p = 0.001) (Table 2) (Fig. 1). A one-way ANOVA analysis was also performed on the four groups (Fig. 4) (F = 14.02; p < 0.0001).

HGF concentration of autistic children with GI disease was compared to GI disease severity (including LNH and erythema). We did not find any significant association between HGF levels and severity of the GI disease, including severity of LNH and erythema, or the presence of autoantibodies (Table 1).

## Discussion

Neuropathological findings in autism indicate altered organization of both the cerebral cortex and cerebellum, both of which are disrupted in mice with decreased MET signaling activity. There is co-occurrence of autism with a number of neurological and cognitive disorders, including epilepsy, atypical sleep patterns, and mental retardation.^30^ Together with well known dysfunction of cortical information processing, the role of MET signaling in interneuron development is relevant as a central component of the hypothesized GABAergic pathophysiological changes in autism.^31^

Although yet to be identified, environmental factors likely contribute to the development of autism, heritability studies suggest that the impact of those factors probably need to be imposed upon individuals genetically predisposed to the disorder.

Individuals with autism can present complex medical profiles, such as gastrointestinal, immune, and nonspecific neurological dysfunctions.^10^–^15^ In addition to brain development, the pleiotropic MET receptor tyrosine kinase has specific roles in digestive system development and repair^18^,^23^,^24^ and modulation of T cell-activated peripheral monocytes and dendritic antigen-presenting cells.^20^,^22^

A polymorphism in the upstream region of the hepatocyte growth factor receptor c-Met, that has been identified in a study involving 1231 autistic children and appropriate controls,^16^ has the dual consequences of a deficiency in c-Met protein level corresponding to reduced transcription rate and down-regulation of (HGF) production, as c-MET/HGF signaling is directly related to HGF expression via a positive feedback mechanism.^43^,^44^ It is thus noteworthy that HGF levels have also been shown to be lower in high functioning autistic adult males.^45^ This raises the question of whether biological activity of HGF contributes to autism symptomatology.

HGF and c-MET both appear to be pertinent beyond brain function and development, since HGF reduces inflammation in other tissues^46^,^47^ and can potently suppress dendritic cell functions.^48^ Thus, deficiencies of both c-MET and HGF lead to conditions that would promote inflammation or oxidative stress,^48^ which would increase the frequency of DNA breakage. Thus, less c-MET could mean less control of inflammation (i.e. more chronic inflammation), more reactive oxygen species, more caspase-mediated damage and more uracil misincorporation.

The neurobiological basis for autism remains poorly understood. Other growth factors, such as transforming growth factor-β1^63^ and Epidermal Growth Factor^64^ have been found to be deficient in autistic patients. Our results show that a significant number of autistic children with severe GI disease have lower concentration of serum HGF when compared to controls, and suggests a relationship between lower HGF levels and GI disease symptoms.

Although hypothetical, deficiency in either HGF or c-MET could initiate and promote a sustained immune response consistent with chronic features of autism pathophysiology, and could therefore be an important contributor to the autism phenotype.

Increased risk for autism, due to a functional polymorphism in the MET gene, and lower levels of unbound HGF, may impart, particularly in individuals with severe GI disease, shared etiology of a parallel, although independent, disruption of brain and peripheral organ development and function. Continued investigations of clinical populations will be needed to determine the contribution of lower concentrations of HGF to the etiology of the complex phenotype of autism.

## Figures and Tables

**Table 1.** Relationship between HGF serum concentration and severity of GI disease.

	**HGF (pg/ml)**	Mean O.D. anti-PR3**	Mean O.D. anti-MPO***	Mean O.D. ASCA****	**Diagnosis A/RA/ASP/PDD**	**LNH**	**Eryth**	**Total GI**	**High LNH**	**Low LNH**	**High erythema**	**Low erythema**	**High total GI**	**Low total GI**	**AutoAbs**	**No AutoAbs**
**Autistic children with GI disease**																
1	535.97	0.129	0.322	0.162	**A**	3	1	6	535.97			535.97	535.97			535.97
2	1416.63	0.121	0.286	0.161	**RA**	1	0	**7**		1416.63		1416.63	1416.63			1416.63
3	251.31	0.197	0.361	0.253	**PDD**	2	1	4		251.31		251.31		251.31		251.31
4	1621.23	0.136	0.284	0.19	**A**	0	0	NA		1621.23		1621.23		1621.23		1621.23
5	847.31	0.099	0.225	0.128	**R-ASP**	2	1	6		847.31		847.31	847.31			847.31
6	847.31	0.175	0.329	0.169	**RA**	3	1	NA	847.31			847.31				847.31
7	1256.51	**0.576**	**0.629**	**0.793**	**A**	**4**	**4**	**11**	1256.51		1256.51		1256.51		1256.51	
8	1710.19	0.245	0.416	0.219	**R-PDD**	1	0	3		1710.19		1710.19		1710.19		1710.19
9	882.90	0.189	0.341	0.169	**A**	1	2	6		882.90		882.90	882.90			882.90
10	713.88	0.366	0.450	**0.556**	**RA**	2	2	6		713.88		713.88	713.88		713.88	
11	776.15	0.134	0.274	0.157	**RA**	2	0	**8**		776.15		776.15	776.15			776.15
12	1060.81	**0.533**	**0.784**	**0.696**	**A**					1060.81		1060.81			1060.81	
13	580.45	**0.544**	**0.504**	0.277	**RA**	3	0	**7**	580.45			580.45	580.45		580.45	
14	838.42	0.227	0.306	0.169	**RA**	3	0	5	838.42			838.42		838.42		838.42
15	527.07	0.289	0.327	0.282	**RA**	**6**	0	**10**	527.07			527.07	527.07			527.07
16	1425.53	0.173	0.394	0.195	**A**	**4**	0	**7**	1425.53			1425.53	1425.53			1425.53
17	687.19	0.166	0.313	0.169	**RA**	3	2	**7**	687.19			687.19	687.19			687.19
18	1185.35	0.141	0.414	0.169	**RA**	2	0	3		1185.35		1185.35		1185.35		1185.35
19	624.92	**0.482**	**0.566**	**0.78**	**RA**	3	2	6	624.92			624.92	624.92		624.92	
20	669.40	0.235	0.435	0.29	**RA**	3	0	6	669.40			669.40	669.40			669.40
21	384.74	0.274	**0.506**	0.343	**R-PDD/NOS**	2	1	4		384.74		384.74		384.74	384.74	
22	509.28	0.221	**0.537**	0.249	**RA**	2	2	**7**		509.28		509.28	509.28		509.28	
23	375.85	0.140	0.356	0.151	**RA**	2	0	4		375.85		375.85		375.85		375.85
24	453.34	0.196	0.360	0.299	**RA**	0	2	5		453.34		453.34		453.34		453.34
25	845.97	0.259	0.463	0.248	**RA**	3	0	4	845.97			845.97		845.97		845.97
26	480.42	0.174	0.457	0.174	**A**	3	**4**	6	480.42		480.42		480.42			480.42
27	660.94	0.199	**0.503**	0.196	**RA**	3	2	5	660.94			660.94		660.94	660.94	
28	800.84	**0.443**	**0.539**	0.426	**R-PDD**	**5**	0	NA	800.84			800.84			800.84	
29	954.28	0.278	**0.529**	0.228	**R-PDD**	3	1	**8**	954.28			954.28	954.28		954.28	
**Mean**	824.97								782.35	870.64	868.46	821.75	805.49	832.73	754.67	861.98
**SD**	376.09								267.12	472.71	548.78	374.94	311.97	520.38	267.13	424.44
									**p = 0.5373**		**p = 0.8690**		**p = 0.8682**		**p = 0.4752**	

HGF concentration of autistic children with GI disease, with (bold) and without LNH, with (bold) and without erythema, with (bold) and without severe total GI disease, and with (bold) and without auto-antibodies (ANCA and/or ASCA), are compared. Diagnosis of autistic children with GI disease (A-autistic; RA-autistic with regressive onset; PDD-pervasive developmental disorder; UD-undetermined; ASP-aspergers), severity of lymphonodular hyperplasia (LNH), presence and severity of erythema, total GI disease severity, as well as the presence of auto-antibodies (AutoAb) are posted. Severity of GI disease was scored according to mild (1 point), moderate (2 points) and marked (3 points) disease in each area (upper and lower GI) and for scope (macroscopic) and histology of each area. Therefore the maximum score for GI disease would be 12 (3 points each for upper scope, upper histology, lower scope and lower histology). A point system was also developed for severity of lymphoid nodular hyperplasia (LNH). Patients were scored according to mild (1 point), moderate (2 points) and marked (3 points) LNH in each area (upper and lower GI) for a maximum of 6 points. And finally, a point system was also developed for severity of erythema. Patients were scored according to mild (1 point), moderate (2 points) and marked (3 points) erythema in each area (upper and lower GI) for a maximum of 6 points.

**Table 2.** Significant difference between HGF in autistic with GI disease and controls.

	**A GI**	**A no GI**	**A GI**	**NA no GI**	**A GI**	**N AGI**
Mean	824.97	1798.23	824.97	2544.65	824.97	1678.37
SD	376.09	1260.76	376.09	1063.49	376.09	694.42
SEM	69.84	380.13	69.84	320.65	69.84	231.47
Count (N)	29	11	29	11	29	9
	p = 0.0005		p = 0.0001		p = 0.001	

Significant difference between HGF concentration (pg/ml) of 29 autistic children with chronic digestive disease (most with ileo-colonic lymphoid nodular hyperplasia (LNH) and inflammation of the colorectum, small bowel and/or stomach) (A GI), and 30 controls (11 age matched autistic children with no GI disease (A No GI), 11 age matched non autistic children without GI disease (NA no GI) and 9 age matched non autistic children with GI disease (NA GI)).

**Table 3.** HGF in autistic vs. controls.

	**Autistic**	**Non autistic**
Mean	1092.619	2154.822
SD	838.3401	996.903
SEM	132.5532	222.9143
N	40	20
p < 0.0001		

Significant difference between HGF concentration (pg/ml) of 40 autistic children (with and without GI disease) and 20 age matched controls (with and without GI disease).

## Footnotes

a The Thoughtful House is a comprehensive treatment and research center, specializing in the care of children with neurological disorders, including autism.

b The Autism Genetic Resource Exchange (AGRE) is the first collaborative gene bank for the study of autism spectrum disorders and one of the world's largest shared resources for the study of autism and related disorders, with a collection of over 900 well-characterized multiplex and simplex families made available to the greater scientific community. Founded by Cure Autism Now (CAN) in 1997, AGRE is currently funded by the National Institute of Mental Health (NIMH) and Autism Speaks (AS), which merged with CAN in 2006.
